# Analysis of Appressorium Formation in *Metarhizium anisopliae* and Its Impact on the Defense Metabolism of *Opisina arenosella* Larvae Based on LC-MS

**DOI:** 10.3390/insects17050476

**Published:** 2026-05-06

**Authors:** Yang Xu, Canxia Wu, Haining Zhang, Dongxu Wang, Huaxin Cai, Hui Wu, Yinghua Tong

**Affiliations:** Forestry College, Fujian Agriculture and Forestry University, Fuzhou 350002, China; xuy0801935@163.com (Y.X.); ktaehyun698@gmail.com (C.W.); 12004290092@fafu.edu.cn (H.Z.); 13526518529@139.com (D.W.); 15080598856@163.com (H.C.); fjwuhui@fafu.edu.cn (H.W.)

**Keywords:** *Metarhizium anisopliae*, appressorium, pathogenicity, cuticular compounds

## Abstract

The appressorium is a specialized infection structure formed by *Metarhizium anisopliae* during host invasion. To investigate the correlation between appressorium formation and fungal pathogenicity, as well as its impact on insect cuticular metabolism, different concentrations of sulforaphane were used to inhibit the appressorium formation rate of *M. anisopliae*. The relationship between appressorium formation rate and pathogenicity against *Opisina arenosella* larvae was evaluated, and LC-MS-based metabolomics was employed to characterize changes in cuticular compounds during the appressorium formation stage, thereby elucidating the chemical responses of the insect cuticle to appressorium formation. The appressorium formation rate of *M. anisopliae* was significantly and positively correlated with its pathogenicity (*p* ≤ 0.05). As the appressorium formation rate increased, pathogenicity against *O. arenosella* larvae increased and the killing speed accelerated. LC-MS metabolomics revealed that after appressorium formation, 102 differential cuticular compounds unique to *O. arenosella* larvae were identified, mainly including benzenes and substituted derivatives, amino acids and derivatives, and heterocyclic compounds. In addition, metabolic pathways associated with immune defense (tyrosine metabolism), antifungal defense (histidine metabolism), and toxin degradation (flavonoid degradation) in the larval cuticle were activated. These results demonstrate that the appressorium plays an important role in host infection by *M. anisopliae*, markedly alters cuticular metabolism, and activates defense- and detoxification-related metabolic pathways in the host. This study provides a theoretical basis for further investigations into fungus–insect cuticle interactions.

## Simple Summary

This study investigates how the appressorium formation of the entomopathogenic fungus *Metarhizium anisopliae* enhances its pathogenicity against *Opisina arenosella* larvae. By inhibiting appressorium formation with sulforaphane, a higher formation rate correlated with increased virulence and faster killing. Post-formation, 102 differential compounds (e.g., L-sorbitol, L-aspartic acid) were detected on the larval cuticle, primarily benzenes, amino acids, and heterocyclic compounds. Concurrently, metabolic pathways for immune defense, antifungal response, and toxin degradation were activated. These findings demonstrate the appressorium’s key role in altering cuticular metabolism and triggering host defense responses, providing a basis for understanding fungus–insect cuticle interactions.

## 1. Introduction

*Metarhizium* is one of the most widely used pathogenic fungi. Studies have shown that *Metarhizium* can parasitize more than 200 species of insects across eight orders and 42 families, as well as infect mites and nematodes [1]. It has demonstrated significant control effects against pests such as *Locusta migratoria manilensis* Meyen [2], *Opisina arenosella* Walker [3], *Hyphantria cunea* Drury [4], and *Brontispa longissima* Gestro [5].

The successful infection of the host by *M. anisopliae* is a complex process. Among the steps, the formation of appressorium is a key stage, where the conidia of the fungus chemically interact with the host’s cuticle to successfully invade. In the interaction between the entomopathogenic fungus and its host, appressorium plays a central role [6]. Appressorium facilitates infection through enzymatic degradation and mechanical pressure exerted by penetration pegs, which help the fungus breach the insect’s cuticle [7]. The formation of appressorium is vital for *M. anisopliae* to penetrate the insect cuticle and colonize within the host [8]. Lü et al. found that appressorium formation is a crucial factor in the virulence of the fungus during infection [6]. Appressorium is rich in mitochondria, Golgi apparatus, endoplasmic reticulum, and ribosomes, and due to its high metabolic activity, these organelles enable, synthesize and secrete a large amount of degrading enzymes such as proteases, lipases, and chitinases [9]. These enzymes play an essential role in exerting mechanical pressure and dissolving the host’s epidermis, thus aiding infection [10,11]. At the tip of the appressorium, a penetration peg grows and secretes epidermal-degrading enzymes, leading to the gradual dissolution of the host’s cuticular wax layer, the formation of honeycomb-like holes, and the destruction of the epidermis. This allows for the breakdown of the insect’s cuticle into nutrients, creating favorable conditions for the fungus to penetrate [12]. Although the key role of appressorium in infection has been confirmed, the mechanism of its formation and its interaction with insect cuticle compounds still require further exploration. This study inhibited the proportion of appressorium formation by regulating sulforaphane concentration, investigated the correlation between conidiophore formation in *M. anisopliae* and its pathogenicity toward *O. arenosella*, and employed liquid chromatography–mass spectrometry (LC-MS) to measure changes in larval cuticular compounds before and after conidiophore formation. These findings provide theoretical support for the pathogenic mechanism of *M. anisopliae* against host insects.

## 2. Materials and Methods

### 2.1. Culturing Media

YEMDT liquid culture medium: Yeast extract 1 g and water 1000 mL, which were autoclaved (0.103 Mpa, 121 °C, 20 min), cooled, and then combined with trehalose (purity: 99.50%, Glpbio, Montclair, CA, USA) to a final concentration of 1 mg/mL.

PPDA solid medium: Peeled potatoes 200 g, glucose 20 g, peptone 10 g, agar 20 g, and water 1000 mL were autoclaved (0.103 Mpa, 121 °C for 20 min).

### 2.2. Insect Hosts

*Opisina arenosella* Walker were collected from Xiang’an District, Xiamen City, Fujian Province (26.969 °N, 118.972 °E). Larvae were maintained in a controlled climate chamber at 26 ± 1 °C and 80% relative humidity and fed with fresh *Washingtonia filifera* palm leaves.

### 2.3. Fungal Strain and Spore Suspension Preparation

The fungal strain used was *Metarhizium anisopliae* (Metschn.) Sorokīn MaHA-01 [3]. After three rounds of subculturing, it was inoculated on PPDA slants and incubated at a constant temperature for spore production. Spores were then collected using sterile water and vortexed for 20 min at 150 rpm to create a spore suspension at a concentration of 1.0 × 10^8^ spores/mL for further use.

### 2.4. Control of Appressorium Formation Rates

Following and modifying the method of Wang et al., sulforaphane (purity: 98.00%, Glpbio, Montclair, CA, USA) was used to inhibit the formation rate of *M. anisopliae* appressorium [13]. Using YEMDT liquid medium, prepare mixtures containing sulforaphane concentrations of 0.00 mg/mL, 0.01 mg/mL, 0.02 mg/mL, 0.05 mg/mL, and 0.08 mg/mL. Prepare spore suspensions with a concentration of 5 × 10^7^ spores/mL using these mixtures. Incubate the suspensions in an incubator at 26 ± 1 °C (150 r/min) in a constant-temperature shaking incubator (ZQTY-50ES, Zhichu, Shanghai, China). At 12 h, 24 h, 36 h, 48 h, and 72 h, 100 μL samples were taken for microscopic examination with an optical microscope (E100, Nikon, Kumagaya City, Japan). Five random fields were examined each time, counting over 100 conidia per field to determine the number of germinated spores and conidia forming appressorium. Each concentration of sulforaphane constituted one treatment, with five replicates per treatment. The germination rate and conidiophore formation rate of *Metarhizium anisopliae* conidia were counted and statistically analyzed.

### 2.5. Pathogenicity Assay of Metarhizium anisopliae with Different Adherent Spore Formation Rates Against Opisina arenosella Larvae

After detecting that different sulforaphane concentrations in 2.5 significantly inhibited the formation rate of adherent cells, a spore suspension with a concentration of 5 × 10^7^ spores/mL was prepared using a mixed solution. Using the immersion method, third-instar larvae of the *O. arenosella* were submerged from the head down into the spore suspension. After 15 s, they were removed, placed on filter paper to air-dry naturally, and transferred into disposable plastic boxes (25 cm × 10 cm × 10 cm). These boxes were placed in an artificial climate chamber maintained at a temperature of (26 ± 1) °C and relative humidity (80 ± 1) % artificial climate chamber and fed with fresh *W. filifera* leaves. Each sulforaphane concentration constituted one treatment, with five replicates per treatment, each containing over 20 test larvae. Larvae immersed in sterile water served as the control (CK). Additionally, a sulforaphane mixture at 0.08 mg/mL in YEMDT liquid medium was used for toxicity verification. After 24 h, larval mortality was observed and recorded daily. Larvae were considered effectively killed when showing no response to brush contact and exhibiting rigor mortis. Dead larvae were placed in a constant-temperature incubator at 26 ± 1 °C to observe *M. anisopliae* growth on the bodies. Then, 48 h post-inoculation, examine five larvae per concentration for conidiophore formation. Using sterile surgical scissors, remove the head and tail, incise along the dorsal–ventral suture, and extract a 5 mm^2^ dorsal abdominal segment. Gently scrape away excess tissue and fat from the inner side of the cuticle with a sterile blade, and spread the cuticle epidermis upward on a sterile microscope slide. Then, add 10 μL of Solution A (containing 10% KOH, 10% glycerol aqueous solution) and 10 μL of Solution B (0.001% Calcofluor White M2R staining solution). After staining for 1 min, rinse with ultrapure water for 3 s and observe the appressorium under a fluorescence microscope [13,14].

### 2.6. Determination of Cuticular Compounds and Metabolites in Larvae of the Opisina arenosella During the Formation of Conidia of Metarhizium anisopliae

#### 2.6.1. Determination of Spore Adhesion Dynamics and Sampling Timing on Larvae

Larvae were inoculated using the immersion method: fourth-instar larvae of *M. anisopliae* were immersed head down in a 1.0 × 10^8^ spores/mL suspension of *O. arenosella* conidia for 15 s, then removed and air-dried on filter paper. Rearing procedures followed those in Section 2.5. At 8 h post-inoculation, randomly select 5 larvae every 2 h to observe spore germination and spore-bearing body formation of *O. arenosella*. Observation methods are the same as in Section 2.5.

#### 2.6.2. Determination of Larval Cuticular Compounds and Metabolism

The inoculation concentration and method were the same as those described in Section 2.6.1. Before and after appressorium formation, ten fourth-instar *O. arenosella* larvae of similar size were randomly selected, submerged in an ice bath in a sterile environment until coma, immersed in 0.1% Triton X-100 aqueous solution (Solarbio, Beijing, China), and gently vortex-rinsed for 10–20 s; then, they were immersed in chromatographically pure hexane (Sigma-Aldrich, Darmstadt, Germany), rinsed for 20–30 s, surface fungal lipids removed, rinsed quickly with ultrapure water, and surface moisture dried with sterile filter paper. Afterwards, they were dried with clean air in the ultra-clean bench [15]. The head and tail were removed with sterile surgical scissors, and the intermediate wall of the abdominal back plate was 5 mm^2^, and the fat body and trachea of the body wall were removed [16]. The samples were placed in 2 mL sterile cryovials, snap-frozen in liquid nitrogen for 1 min, ground, and stored for subsequent analysis. Five biological replicates were prepared for each of the pre- and post-appressorium formation groups, with a total of 50 larvae per treatment. Larvae immersed in sterile water for the same duration served as the control.

Cuticular compounds of *O. arenosella* were analyzed using a Thermo Fisher Scientific Vanquish UHPLC system (Thermo Fisher Scientific, Dreieich, Germany) coupled with an Orbitrap Exploris 120 mass spectrometer (Thermo Fisher Scientific, Germany). Chromatographic separation was performed on a Waters ACQUITY UPLC BEH Amide column (1.8 μm, 2.1 × 100 mm) to characterize changes in cuticular compounds of *O. arenosella* before and after appressorium formation by *M. anisopliae*. Briefly, 100 mg of liquid-nitrogen-ground sample was transferred into an EP tube; then, 500 μL of 80% methanol (LC-MS Garde, CNW Technologies, Düsseldorf, Germany) aqueous solution was added, before the sample was vortexed, and incubated on ice for 5 min, followed by centrifugation at 4 °C and 15,000× *g* for 20 min using a refrigerated centrifuge (D3024R, Scilogex, Rocky Hill, CT, USA). The supernatant was collected and diluted with LC-MS-grade water to adjust the methanol content to 53%, then centrifuged again under the same conditions for 20 min. The resulting supernatant was collected for LC-MS analysis. In addition, equal volumes of each sample were pooled to prepare a quality control (QC) sample [17].

LC analysis was conducted in both positive- and negative-ion modes with a column temperature of 40 °C and a flow rate of 0.2 mL/min. In positive-ion mode, mobile phase A was 0.1% formic acid (LC-MS Garde, CNW Technologies, Düsseldorf, Germany) and mobile phase B was methanol; in negative-ion mode, mobile phase A was 5 mM ammonium acetate (LC-MS Garde, SIGMA-ALDRICH, St. Louis, MO, USA, The United States of America, pH 9.0) and mobile phase B was methanol. The gradient elution program was as follows: 0–1.5 min, 2% B; 1.5–3.0 min, 85% B; 3.0–10.0 min, 100% B; 10.0–10.1 min, 2% B; 10.1–11.0 min, 2% B; and 11.0–12.0 min, 2% B.

MS conditions were as follows: scan range *m*/*z* 100–1500 with an electrospray ionization (ESI) source. The parameters were set to a spray voltage of 3.5 kV, sheath gas flow rate of 35 psi, auxiliary gas flow rate of 10 L/min, ion transfer tube temperature of 320 °C, RF lens level of 60, and auxiliary gas heater temperature of 350 °C. Data were acquired in both positive- and negative-ion modes [18].

### 2.7. Data Statistical Analysis

Data were organized using Excel 2016 and statistically analyzed with SPSS 27.0 software. Probit regression was first applied to the dose–mortality data to fit the virulence regression equation, yielding the median lethal time (LT_50_), median lethal concentration (LC_50_), and correlation coefficient, R. Duncan’s multiple range test was employed for multiple comparisons. Data on the germination of *M. anisopliae* conidia on *O. arenosella* carcasses were processed in Excel and plotted as scatter plots using Origin 2024. Raw LC-MS data were acquired using Compound Discoverer 3.3 and underwent data processing including peak extraction and alignment. Metabolite identification was performed using mzCloud, mzVault, and Masslist databases for mass spectrometry peak recognition. Identified metabolites were annotated using KEGG, HMDB, and IPIDMaps databases. For multivariate statistical analysis, metabolomics data were processed with metaX software (v1.6.0) to perform PCA and OPLS-DA analyses, yielding VIP values for each metabolite. Univariate analysis calculated *p*-values and fold change (FC) values based on *t*-tests. Differentially expressed metabolites were screened using criteria: VIP > 1, *p* < 0.05, and FC ≥ 2 or FC ≤ 0.5. Volcano plots were generated using the R package ggplot2 (v3.5.1), combining VIP, log2(FC), and −log10(P) to identify target metabolites. Pearson correlation coefficients for differential metabolites were calculated using R functions, with significance tested at *p* < 0.05. Correlation plots were visualized with the corrplot package. Then, bubble plots were generated using ggplot2. Metabolic pathways were analyzed using the KEGG database, with enrichment determined by x/n > y/n and *p* < 0.05 indicating significant enrichment.

## 3. Results

### 3.1. Inhibited Effect of Different Sulforaphane Concentrations on Metarhizium anisopliae Germination Rate

**Figure 1:**
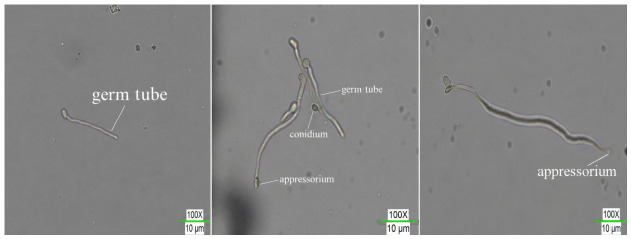
Germ tube and appressoria after conidia germination of *Metarhizium anisopliae* in liquid fermentation.

The morphology of germ tube and appressoria after conidia germination of *Metarhizium anisopliae* in liquid fermentation broth is shown in Figure 1. Statistical analysis of *M. anisopliae* conidial germination rate and appressorium formation rate under different sulforaphane concentrations is presented in Supplementary Table S1. As shown in Supplementary Table S1, both conidial germination rate and appressorium formation rate decreased with increasing sulforaphane concentration. For conidia germination rate, the 0 mg/mL treatment exhibited significantly higher germination rates (*p* ≤ 0.05) than other treatments at all time points. At 36 h, the germination rate reached (92.40 ± 1.34) %, significantly higher (*p* ≤ 0.05) than other treatments, after which the rate stabilized. At 72 h, germination rates exceeded 80.00% across all treatments. Regarding appressorium, the formation rate under the 0 mg/mL treatment was significantly higher (*p* ≤ 0.05) than other treatments at all time points. At 72 h, a significant dose–response gradient (*p* ≤ 0.05) was observed in appressorium formation rates among treatments, with the 0 mg/mL treatment exhibiting a formation rate of (66.60 ± 1.67) %, significantly higher (*p* ≤ 0.05) than the other four groups. As sulforaphane concentration increased, the appressorium formation rate decreased significantly (*p* ≤ 0.05). These results indicate that sulforaphane at certain concentrations can significantly inhibit (*p* ≤ 0.05) the formation of *M. anisopliae* appressorium.

### 3.2. Pathogenicity of Metarhizium anisopliae with Different Appressorium Formation Rates on Opisina arenosella Larvae

**Figure 2:**
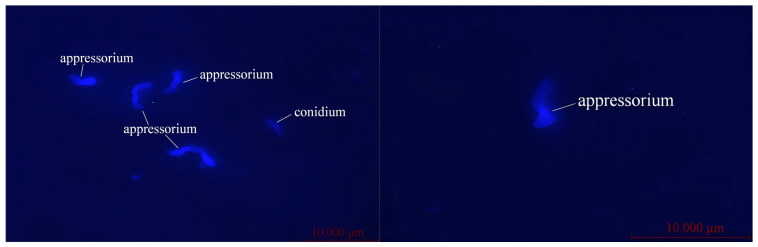
Fluorescent staining of conidium and appressoria of *Metarhizium metarhizium* in the larvae.

Fluorescently stained microscopic images of *Metarhizium anisopliae* conidia and appressoria in the larvae are shown in Figure 2. After inhibiting the appressorium formation rate with different concentrations of sulforaphane, statistical analysis was performed on the pathogenicity of *M. anisopliae* to *O. arenosella* larvae, and the results are shown in Supplementary Table S2. From Supplementary Table S2, it can be observed that when the appressorium formation rate of *M. anisopliae* was higher (sulforaphane concentration of 0 mg/mL), the cumulative corrected mortality of coconut webworm larvae at 5 days and 7 days was significantly higher (*p* ≤ 0.05) than that of other treatments. The cumulative corrected mortality at 7 days was 82.76%, with the LT_50_ being smaller at 4.82 days. When the appressorium formation rate was lower (sulforaphane concentration of 0.08 mg/mL), the mortality rate of coconut webworm larvae was the lowest, with a cumulative corrected mortality of 25.29% at 7 days, which was significantly lower (*p* ≤ 0.05) than other treatments, and the LT_50_ was larger at 8.74 days. Under the treatment of YEMDT and sulforaphane mixed solution, there was no significant difference in the mortality rate of *O. arenosella* larvae compared to the control (CK), indicating that this mixed solution was non-toxic to *O. arenosella* larvae. Therefore, it can be concluded that the appressorium formation rate of *M. anisopliae* is positively correlated with its pathogenicity to insects. The higher the appressorium formation rate, the stronger its pathogenicity.

### 3.3. Dynamic of Appressorium Formation on Insect Bodies and Sampling Time Analysis

**Figure 3:**
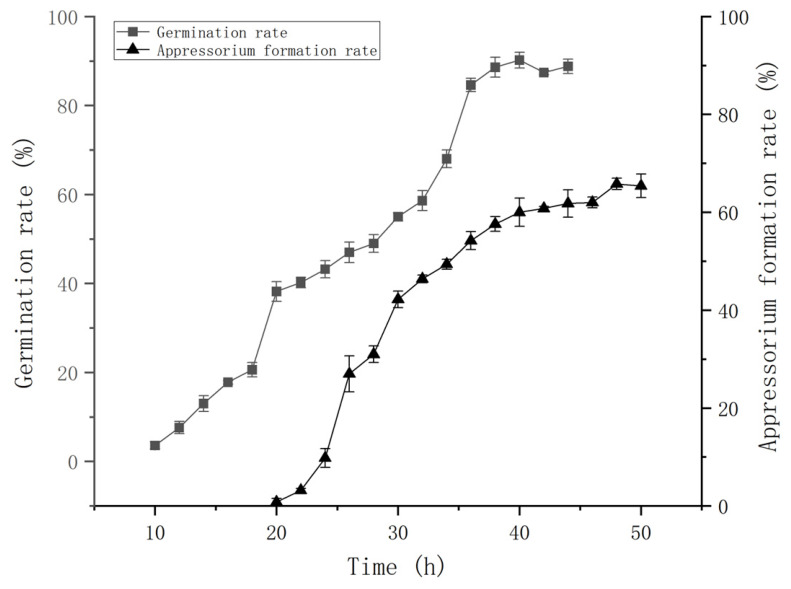
The germination rate and appressorium formation rate of sporangia of *Metarhizium anisopliae* on the larvae of *Opisina arenosella* changed dynamically.

Statistical analysis was performed on the germination of conidia and the formation of appressorium on the body wall of *O. arenosella* larvae infected with *M. anisopliae*. The results are shown in Figure 3. From the figure, it can be observed that 10 h after inoculation, conidia began to germinate, with a germination rate of 3.60%. Over time, at 38 h, the germination rate significantly increased to 90.40%, and then stabilized. At 20 h, the germinated conidia began to form appressorium. From 22 to 30 h, the process of appressorium formation accelerated significantly. At 48 h, the appressorium formation rate reached 65.40%, and then stabilized. From the results, it can be concluded that the sampling time before appressorium formation was 18 h post-inoculation, while the sampling time after appressorium formation was 48 h post-inoculation.

### 3.4. Analysis of the Effects of Appressorium Formation by Metarhizium anisopliae on the Cuticular Metabolites of Opisina arenosella Larvae

#### 3.4.1. Orthogonal Partial Least Squares Discriminant Analysis (OPLS-DA) Results

**Figure 4:**
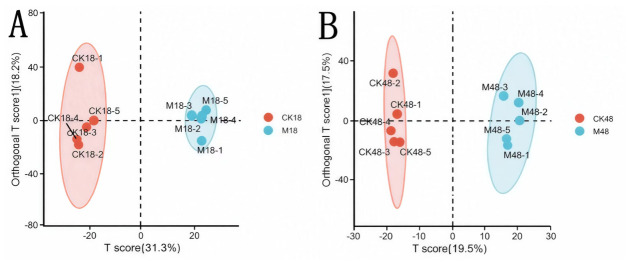
The OPLS-DA score diagram of the body wall compounds of *Opisina arenosella* larvae before and after the formation of *Metarhizium* appressorium. (**A**) Before appressorium formation. (**B**) After appressorium formation. Note: The x-axis shows the scores of the predictive component. Differences along this axis correspond to systematic variation between groups. The y-axis displays scores of the orthogonal component, where spread along this axis captures variation within groups. The percentages shown beside each axis indicate how much of the total variance in the dataset is explained by that component. Model predictive performance is measured using Q^2^: a model is considered statistically valid when Q^2^ > 0.5.

The OPLS-DA results revealed significant metabolic differences among the treatment groups. Specifically, the OPLS-DA model comparing the pre-appressorium formation group with the time-matched control showed excellent performance parameters (R^2^X = 0.495, R^2^Y = 0.991, Q^2^Y = 0.848). The two groups were clearly separated along the t1 principal component (31.30%), indicating the strong regulatory effect of the experimental treatment on metabolite composition (Figure 4A). In contrast, although the OPLS-DA model comparing the post-appressorium formation group with the time-matched control also exhibited good fit (R^2^X = 0.370, R^2^Y = 0.993, Q^2^Y = 0.657), the separation along the t1 principal component (19.5%) was relatively weaker (Figure 4B). This suggests that the metabolic changes between these two groups may be influenced by a more complex biological regulatory network, or that the magnitude of metabolic alteration was relatively small. Overall, all treatment groups showed distinct metabolic profiles compared with the control group. Moreover, across all OPLS-DA models, R^2^Y values were close to 1 and Q^2^Y values exceeded the critical threshold of 0.5, supporting the reliability and stability of the analysis.

#### 3.4.2. Differential Metabolite Analysis

**Figure 5:**
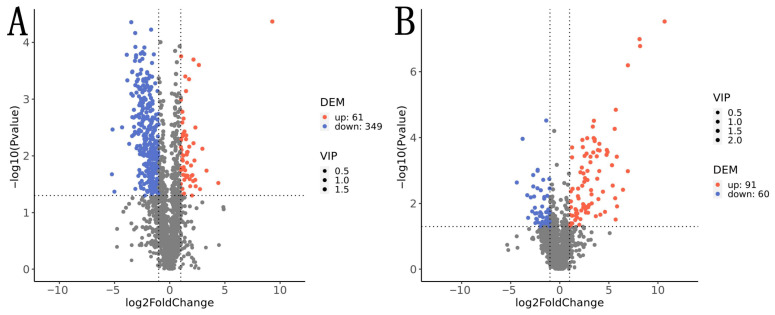
Volcano plots of *Opisina arenosella* larva body wall compounds before and after the formation of *Metarhizium* appressorium. (**A**) Before appressorium formation. (**B**) After appressorium formation. Note: In the volcano plot, each point represents a metabolite. Metabolites that are significantly upregulated are shown in red, while those significantly downregulated are shown in blue. The size of each symbol is proportional to its Variable Importance in Projection (VIP) score. The x-axis represents the log_2_-transformed fold change, where larger absolute values indicate greater differences in abundance between the experimental groups. The y-axis displays −log_10_ (*p*-value), with higher values corresponding to greater statistical significance of differential expression.

Differential metabolite analysis showed that, compared with the time-matched control, 410 differential cuticular metabolites were detected before appressorium formation. The number of downregulated metabolites (349) was far greater than that of upregulated metabolites (61), indicating that *Metarhizium anisopliae* mainly exerted an inhibitory effect on the host cuticular metabolic network prior to appressorium formation. Representative upregulated metabolites included pheophorbide a, urobilin, and 4-chloro-2-nitrobenzyl alcohol. In contrast, representative downregulated metabolites included 27-hydroxycholesterol, 1-methylxanthine, and valeryl-4-hydroxyvalsartan. These changes were mainly associated with pathways related to caffeine metabolism and porphyrin metabolism (Figure 5A). Compared with the time-matched control, 151 differential metabolites were detected after appressorium formation, including 91 upregulated and 60 downregulated metabolites. The markedly upregulated cuticular metabolites included the secondary metabolites epigallocatechin gallate, spermidine, and glycyl-L-phenylalanine, whereas the significantly downregulated metabolites included fumonisin B2, manghaslin, and GDP-L-fucose (Figure 5B).

#### 3.4.3. Venn Diagram of Differential Compounds in the Larval Cuticle Before and After Appressorium Formation by *Metarhizium anisopliae*

**Figure 6:**
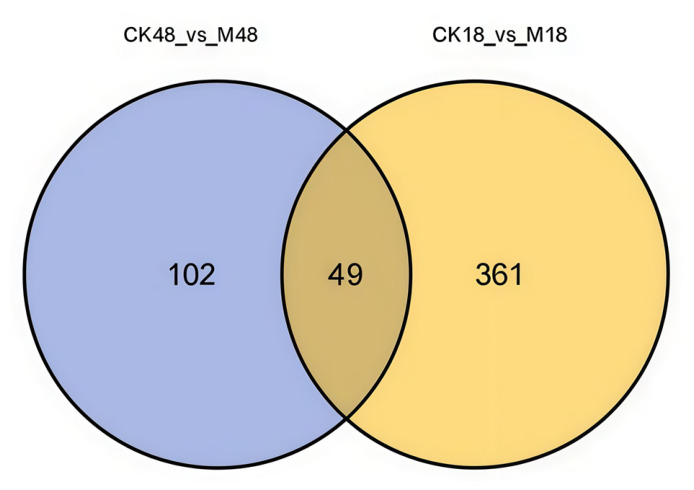
Venn diagram of the differential compounds in the body wall of the larvae before and after the formation of *Metarhizium* appressorium. Note: M18, inoculated group before appressorium formation (18 h); CK18, control group before appressorium formation (18 h); M48, inoculated group after appressorium formation (48 h); CK48, the control group after appressorium formation (48 h).

A Venn diagram was used to compare the differential cuticular compounds in *O. arenosella* larvae before and after appressorium formation by *M. anisopliae* (Figure 6). As shown in Figure 6, 410 differential cuticular compounds were detected before appressorium formation, whereas 151 were detected after appressorium formation. Among them, 361 differential cuticular compounds were unique to the pre-appressorium stage, and 102 were unique to the post-appressorium stage (see Supplementary Table S3 for details), while 49 differential cuticular compounds were shared between the two stages.

#### 3.4.4. Categories of Differential Cuticular Compounds Unique to *Opisina arenosella* Larvae After Appressorium Formation

**Figure 7:**
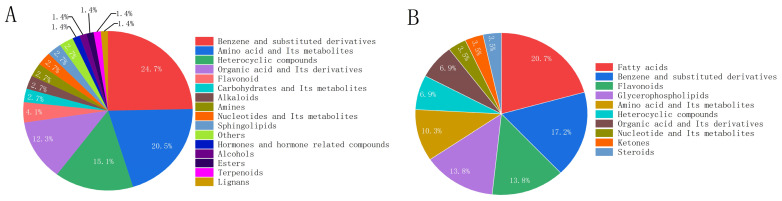
Types of compounds unique to the body wall of *Opisina arenosella* larvae after the formation of *Metarhizium* appressorium. Note: (**A**) Significantly up-regulated compounds. (**B**) Significantly down-regulated compounds.

The categories of differential cuticular compounds unique to the post-appressorium stage were statistically analyzed, and the results are shown in Figure 7. As shown in Figure 7, 73 cuticular compounds in *O. arenosella* larvae were significantly upregulated, including L-sorbitol, sparfloxacin, N-Acetyl-D-glucosamine, L-Aspartic acid, and 2,6-dihydroxybenzoic acid. These upregulated compounds mainly belonged to benzenes and substituted derivatives, amino acids and derivatives, and heterocyclic compounds, with benzenes and substituted derivatives accounting for 24.7%.

In addition, 29 cuticular compounds were significantly downregulated, including lincomycin, D-Gulono-1,4-lactone, luteolin, manghaslin, and 4,5-dihydroxyterephthalic acid. These downregulated compounds were mainly fatty acids, benzenes and substituted derivatives, and flavonoids, with fatty acids accounting for 20.7%.

#### 3.4.5. KEGG Enrichment Analysis of Cuticular Compounds in *Opisina arenosella* Larvae During *Metarhizium anisopliae* Infection

**Figure 8:**
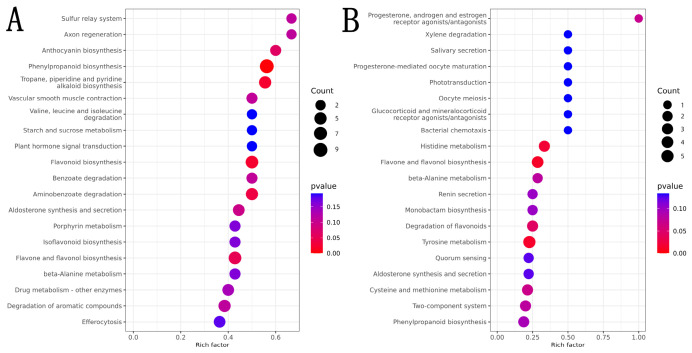
Bubble plot of metabolic pathways for cuticular compounds in *Opisina arenosella* larvae before and after appressorium formation by *Metarhizium anisopliae*. (**A**) Before appressorium formation. (**B**) After appressorium formation.

KEGG pathway enrichment analysis revealed significant differences in metabolic pathway regulation among the treatment groups. Compared with the time-matched control, 152 metabolic pathways were annotated before appressorium formation, among which 25 pathways showed significant differences (*p* ≤ 0.05) and 9 showed highly significant differences (*p* ≤ 0.01). The pathways with pronounced differences and relatively large numbers of differential metabolites included Tropane, piperidine and pyridine alkaloid biosynthesis, phenylpropanoid biosynthesis, and flavonoid biosynthesis (Figure 8A). These results indicate that the host rapidly synthesizes alkaloids, flavonoids, and phenylpropanoids with antifungal, antioxidant, and signaling functions by enhancing the activity of these defensive secondary metabolic pathways, thereby systemically resisting *M. anisopliae* infection and spread.

Compared with the time-matched control after appressorium formation, 101 metabolic pathways were annotated, among which 15 pathways showed significant differences (*p* ≤ 0.05). The major affected pathways included tyrosine metabolism, histidine metabolism, and degradation of flavonoids (Figure 8B). This suggests that, following appressorium formation by *M. anisopliae*, the host activates these key metabolic pathways to strengthen immune defense, antifungal responses, and toxin degradation mechanisms, thereby systemically resisting appressorium-mediated infection.

## 4. Discussion

In the infection process of *M. anisopliae*, the appressorium plays a crucial role. The appressorium is rich in mitochondria, Golgi apparatus, endoplasmic reticulum, and ribosomes, and these organelles enable the fungus to exert concentrated mechanical force and enzymatic activity on the host epidermis, thereby effectively penetrating host tissues and laying the foundation for subsequent infection [19]. Sulforaphane is a natural isothiocyanate compound, mainly found in cruciferous vegetables, such as broccoli, cabbage, and cauliflower. Research has confirmed that sulforaphane can exert antibacterial effects through multiple mechanisms, including inhibiting bacterial virulence factors, reducing biofilm formation, and lowering bacterial oxidative stress tolerance [20]. Sulforaphane can not only significantly inhibit the growth of Candida albicans, but also suppress its biofilm formation and hyphal development [21]. In addition, sulforaphane can also inhibit the germination of *Beauveria bassiana* conidia and the formation of appressoria, significantly reducing the pathogenicity of *Beauveria bassiana* to *Solenopsis invicta* [22]. In this study, sulforaphane concentration was found to significantly inhibit the appressorium formation rate of *M. anisopliae*. Moreover, the appressorium formation rate was positively correlated with fungal pathogenicity: a higher appressorium formation rate resulted in stronger virulence and a shorter lethal time.

Meng reported that deletion of ribonuclease 1 made it difficult for *M. anisopliae* to obtain sufficient nutrients from the external environment for growth and development, thereby delaying appressorium formation. Under nutrient-limited conditions, *M. anisopliae* cannot absorb enough nutrients to meet the requirements for appressorium formation; consequently, appressorium formation is postponed, leading to a significant reduction in pathogenicity [23]. Duan et al. mutated the phosphorylation-site threonine residues in the *M. anisopliae MPL1* gene to alanine and isoleucine, which decreased the appressorium formation rate and consequently reduced insecticidal virulence [24]. Meng also found that deletion of the *M. robertsii Ste12-*like transcription factor (MrSt12) abolished appressorium formation and completely eliminated pathogenicity [25]. Meng demonstrated that appressorium formation is essential for *Magnaporthe oryzae* invasion of rice; after deletion of *Mo WHI2* and *Mo PSR1*, appressorium function became abnormal, the proportion of penetration peg formation decreased significantly, and invasive hyphal expansion was slowed [26]. He et al. altered the turgor pressure of *Exserohilum turcicum* during appressorium development and maturation using tricyclazole and found that host infection efficiency was also markedly reduced [27]. Collectively, these findings indicate that regulating appressorium formation and function can effectively influence the infection capacity of pathogenic fungi, thereby providing new ideas and strategies for the control of agricultural diseases.

Our results showed that after appressorium formation, several metabolic pathways were significantly enriched, including histidine metabolism, tyrosine metabolism, and flavonoid degradation. Specifically,

(1) Tyrosine metabolism is closely associated with immune defense mechanisms. Previous studies have shown that tyrosine metabolism in *Lymantria dispar* larvae changed markedly after viral infection, which was linked to the upregulation of genes related to energy metabolism and the nervous system [28]. This suggests that tyrosine metabolism is involved in behavioral regulation in insects following infection.
(2) In the insect immune system, histidine metabolism contributes to antifungal defense through multiple mechanisms. Histidine-derived metabolites (e.g., histamine) act as signaling molecules in immune responses and can activate insect immune defense mechanisms [29]. These metabolites may also function as signals to induce the expression of antifungal peptide genes, thereby enhancing antifungal capacity [30]. In addition, imidazoleacetic acid, a histidine metabolite, exhibits broad-spectrum antimicrobial activity in Lepidopteran insects (e.g., the silkworm). The expression of the key enzyme histidine decarboxylase in its biosynthetic pathway increases by 3–5 fold after hemolymph infection [31]. Studies in *Drosophila melanogaster* further showed that individuals lacking histidine kinase exhibited a >40% reduction in their ability to clear Gram-negative bacteria [31].
(3) The flavonoid degradation pathway is crucial for insect life activities, as many flavonoids exert insecticidal or antifeedant effects [32]. For example, flavonoids in cotton, such as rutin, quercetin, and isoquercitrin, inhibit the growth and pupation of *Helicoverpa armigera*. These compounds reduce insect growth and development by altering feeding behavior and decreasing plant consumption [33]. Flavonoids can also inhibit the activity of insect digestive enzymes, thereby impairing food utilization efficiency. For instance, tannin compounds can bind to proteins to form stable cross-linked complexes, which suppress digestive enzyme activity and reduce nutrient absorption in insects [34]. Insects possess metabolic enzymes capable of metabolizing and degrading flavonoids. For example, the flavonoid reductase from the gut bacterium *Flavonifractor plautii* can initiate the degradation of flavones and flavonols. In addition, certain strains of *Lactococcus* and *Enterococcus* exhibit deglycosylation activity toward specific C- and O-glycosides of flavonoids, causing structural modifications that reduce toxicity [35]. Moreover, some insects can enhance flavonoid metabolism and detoxification capacity by upregulating the expression of detoxification enzymes in vivo [36].

Our results showed that the cuticular compounds of *O. arenosella* larvae differed markedly from those of the control group both before and after appressorium formation by *M. anisopliae*. After appressorium formation during *M. anisopliae* infection, 102 unique differential cuticular compounds were identified, mainly including benzenes and substituted derivatives, amino acids and derivatives, and heterocyclic compounds. Several of these compounds merit further discussion:

(1) Sparfloxacin exhibits certain antifungal activity. Au-aff et al. reported that sparfloxacin could inhibit the growth of *Candida albicans* and *Aspergillus niger* [37].
(2) 2,6-Dihydroxybenzoic acid, also known as gentisic acid, is a bioactive compound with multiple biological functions. Its molecular formula is C_7_H_6_O_4_, and its structure contains two phenolic hydroxyl groups and one carboxyl group, which confer pronounced antioxidant properties and potential antimicrobial activity. Although studies on its antifungal effects remain limited, evidence from related analogs is available. For example, Hu et al. reported that 2,5-dihydroxybenzoic acid, one of the active components in quinoa seeds, possesses certain antifungal properties [38].
(3) Sulfadoxine competitively inhibits dihydropteroate synthase, blocking the condensation of p-aminobenzoic acid with pteridine pyrophosphate to form dihydropteroate. This inhibits folate biosynthesis, preventing bacteria and fungi from synthesizing DNA due to folate deficiency, ultimately leading to death [39,40].

During *M. anisopliae* infection of host insects, the chemical interactions with the host cuticle are highly complex. Cuticular compounds can provide nutrients for *M. anisopliae* during infection, while also exerting chemical defense effects or acting as chemical cues that induce fungal responses. In this study, we preliminarily investigated changes in cuticular compounds of *O. arenosella* larvae during appressorium formation by *M. anisopliae*; however, the functional roles and underlying mechanisms of these compounds in the appressorium formation process remain to be further validated.

## 5. Conclusions

This study found that (1) the insect body wall is not a passive barrier, but can dynamically respond to *Metarhizium anisopliae* infection through metabolome reprogramming. (2) The interaction between *Metarhizium anisopliae* and the cuticle wall of host insects involves not only mechanical and enzymatic hydrolysis processes, but also complex chemical signals and metabolic disturbances.

This study established a new framework of fungal–insect body wall interaction, which provided a new theoretical basis for elucidating the pathogenic mechanism of *Metarhizium anisopliae* and optimizing its biological control application. Further research is needed to reveal the molecular pathways of body wall metabolites regulating appressorium development and fungal pathogenicity.

## Data Availability

The original contributions presented in this study are included in the article/Supplementary Materials. Further inquiries can be directed to the corresponding author.

## Supplementary Materials

The following supporting information can be downloaded at https://www.mdpi.com/article/10.3390/insects17050476/s1, Table S1: Spore germination rate and appressorium formation rate of *Metarhizium anisopliae*; Table S2: Different appressorium formation rates of *Metarhizium anisopliae* and their pathogenicity to *Opisina arenosella*; Table S3: Compounds specific to the *Opisina arenosella* larva Body Wall after the formation of *Metarhizium appressorium*.
