# Supplementary material for: Analysis of Appressorium Formation in Metarhizium anisopliae and Its Impact on the Defense Metabolism of Opisina arenosella Larvae Based on LC-MS
Source: Insects. 2026 May 6;17(5):476. doi: 10.3390/insects17050476 (PMC13207983; doi:10.3390/insects17050476)
Supplement: Supplementary file 1 [file insects-17-00476-s001.zip › insects-4216685-supplementary.pdf]

Table S1 Spore germination rate and appressorium formation rate of *Metarhizium anisopliae*

Sulforaphane Concentration （mg/mL）	12h germination rate （%）	24h germination rate （%）	24h appressorium germination rate （%）	36h germination rate （%）	36h appressorium germination rate （%）	48h germination rate （%）	48h appressorium germination rate （%）	72h germination rate （%）	72h appressorium germination rate （%）
0	37.2±3.27a	60.60±4.39a	24.60±1.52a	92.40±1.34a	47.60±3.13a	90.60±2.07a	62.20±1.48a	91.40±1.67a	66.60±1.67a
0.01	8.00±0.84b	32.40±4.93b	2.80±0.84b	64.00±2.74b	47.60±3.13b	83.40±1.67b	50.60±3.42b	87.80±1.14b	56.60±1.52b
0.02	3.00±1.22b	5.40±0.89c	0.00±0.00c	32.60±1.82c	32.20±1.92c	79.40±1.82c	48.00±1.14b	86.80±0.55b	52.50±1.67c
0.05	0.00±0.00c	4.20±1.30c	0.00±0.00c	29.80±2.28d	10.40±1.34d	59.40±1.34d	38.20±1.58c	82.40±0.89b c	41.80±0.84d
0.08	0.00±0.00c	0.60±0.00d	0.00±0.00 c	12.40±1.14e	6.60±1.14e	48.60±2.07e	23.80±1.92d	81.60±1.41c	29.60±2.19e

Note: The data in the table are the average results obtained from five repeated experiments, expressed as "average ± standard deviation". If different letters are marked on the data, it indicates that there are significant differences between these data ($p \leq 0.05$); if the same letters are marked, it means that there is no significant difference between the data in the same column ($p > 0.05$).

Table S2 Pathogenicity of different appressorium formation rates of *Metarhizium anisopliae* to *Opisina arenosella*

Tested insects	Sulforaphane concentration （mg/mL）	48h appressorium germination rate	5d Cumulative mortality （%）	5d Cumulative corrected mortality （%）	7d Cumulative mortality （%）	7d Cumulative corrected mortality （%）	Mycosis rate （%）	Correlation coefficient R^2	Lethal Time 50% （d）	Regression equation
Opisina arenosella	0	65.20±2.86a	51.00±1.30a	44.94±1.56a	85.00±2.35a	82.7	89.00	0.971	4.82	y=11.75x-12.40
	0.01	63.40±2.70ab	36.00±2.95b	28.09±0.23b	65.00±3.08b	59.77	85.00	0.954	6.23	y=19.78x-14.07
	0.02	59.40±1.52b	30.00±2.35bc	21.35±0.51c	66.00±1.48b	60.92	79.00	0.915	6.37	y=10.49x-19.20
	0.05	47.00±2.55c	28.00±2.19bc	19.10±0.56c	60.00±1.58b	54.02	80.00	0.906	6.85	y=9.15x-15.00
	0.08	39.60±1.95d	16.00±1.92bd	6.25±0.41d	35.00±2.00c	25.29	65.00	0.961	8.74	y=7.08x-12.00
	CK	/	11.00±1.30d	/	13.00±1.52d	/	/	/	/	/
	Non-toxic verification	/	14.00±1.64d	/	15.00±1.41d	/	/	/	/	/

Note: The data in the table are the average results obtained from five repeated experiments, expressed as "average ± standard deviation". If different letters are marked on the data, it indicates that there are significant differences between these data ($p \leq 0.05$); if the same letters are marked, it means that there is no significant difference between the data in the same column ($p > 0.05$).

Table S3 Compounds specific to the *Opisina arenosella* larva Body Wall after the formation of *Metarhizium* appressorium（"CK" represents the control group at the same time and "After appressorium formation" indicates that the appressorium formation rate tends to be stable.）

Metabolin	Category	m/z	RT/min	Relative conte		variety
				CK	After appressorium formation	
L-Iditol	Alcohols	227.0747	1.8	2.44	708.776	↑
Epinephrine	Hormones and hormone related compounds	182.0824	1.8	6.356	1778.918	↑
3alpha,4,7,7alpha-Tetrahydro-4-hydroxy-1H-isoindole-1,3（2H）-dione	Heterocyclic compounds	226.0719	1.8	0.768	1260.388	↑
Sparfloxacin	Benzene and substituted derivatives	393.1652	1.9	5.64	692.13	↑
N-Acetyl-D-glucosamine	Carbohydrates and Its metabolites	221.0926	1.7	38.604	1865.848	↑
Penciclovir	Heterocyclic compounds	253.1187	1.7	27.794	811.058	↑
Porphobilinogen	Organic acid and Its derivatives	225.0955	2.7	26.15	711.036	↑
Sulindac sulfone	Benzene and substituted derivatives	373.0985	7.7	3990.292	9491.486	↑
Trp-Cys	Amino acid and Its metabolites	272.0918	2.3	8.712	488.486	↑
1-Oleoyl-2-palmitoyl-sn-glycero-3-phosphocholine	Glycerophospholipids	760.583000000001	6.6	34161.472	13119.848	↓
N-Benzyloxycarbonylglycine	Benzene and substituted derivatives	192.0652	3.8	17.14	685.364	↑
Salsolinol	Heterocyclic compounds	224.0918	2.7	22.682	1185.974	↑
Gly-Trp	Amino acid and Its metabolites	350.0805	3.7	22480.546	246606.964	↑
Guanosine 3',5'-Cyclic Monophosphate	Nucleotide and Its metabolites	344.0427	1.1	1004.918	4953.516	↑
N-Acetyl-L-tyrosineN	Amino acid and Its metabolites	222.0766	2.6	775.208	8638.134	↑
2,7-Dihydroxy-5-methyl-1-naphthoic acid	Benzene and substituted derivatives	263.0487	3.6	1133.846	13291.514	↑
5,5'-Dehydrodivanillate	Benzene and substituted derivatives	352.0961	3.9	1133.846	13291.514	↑
Sulfa aciddoxine	Benzene and substituted derivatives	309.0725	2.4	2750.09	29218.008	↑
Cys-Trp	Amino acid and Its metabolites	306.0906	3.7	4217.934	43439.758	↑
Austidiol	Alcohols	235.0543	2.7	2040.322	14443.344	↑
D-Gulono-1,4-lactone	Esters	223.054	2.1	1023.686	16773.56	↑
Isofraxidin	Heterocyclic compounds	281.0598	2.7	1010.906	8809.906	↑
Dapsone hydroxylamine	Benzene and substituted derivatives	323.0706	3.6	1250.634	5938.118	↑
1,1,1,2,2-Pentafluoro-7-phenylheptan-3-one	Benzene and substituted derivatives	279.0802	3.6	1225.196	16688.966	↑
6-Acetylmorphine	Alkaloids	386.1702	3.0	1446.28	9596.07	↑
Normetanephrine	Benzene and substituted derivatives	228.0775	2.0	13.594	1179.624	↑
h-gamma-Glu-leu-oh	Amino acid and Its metabolites	259.1288	2.4	23378.252	1694.58	↓
Vanillylmandelic acid	Benzene and substituted derivatives	395.0962	2.3	7598.05	38671.714	↑

Table S3 Continuation

Metabolin	Category	m/z	RT/min	Relative conte		variety
				CK	After appressorium formation	
Methiothepin	Heterocyclic compounds	374.1703	2.0	9.89	415.858	↑
Parecoxib	Benzene and substituted derivatives	371.1006	7.9	12950.706	30903.684	↑
Irsogladine	Benzene and substituted derivatives	254.9938	2.4	7538.516	1153.638	↓
Cinnamoylglycine	Amino acid and Its metabolites	264.0808	2.1	1259.014	6160.974	↑
4-Methylhippuric acid	Benzene and substituted derivatives	192.0666	2.7	1420.862	39392.986	↑
5-Hydroxyindoleacetic acid	Organic acid and Its derivatives	190.0509	3.7	1802.238	10399.418	↑
gamma-Glutamylglutamate	Amino acid and Its metabolites	311.0719	2.4	1419.712	12429.144	↑
Valylcysteine	Amino acid and Its metabolites	219.0767	2.0	877.71	14624.93	↑
Isoleucyl-threonine	Amino acid and Its metabolites	341.1345	1.5	735.844	10878.57	↑
4-Hydroxy-3-methoxy cinnamaldehyde	Benzene and substituted derivatives	196.0965	2.7	2291.924	12238.656	↑
Asp-Trp	Amino acid and Its metabolites	426.0775	2.9	1601.762	6887.986	↓
[(2S,3R,4S,5S,6R)-3,4,5-trihydroxy-6-(hydroxymethyl)oxan-2-yl] (1Z)-N-sulfooxyheptanimidothioate	Others	402.0906	2.8	709.032	4196.02	↑
Citrinin	Heterocyclic compounds	249.0697	3.5	2573.23	36267.004	↑
3-[3-Tert-butylsulfanyl-1-[(4-chlorophenyl)methyl]-5-propan-2-ylindol-2-yl]-2,2-dimethylpropanoic acid	Organic acid and Its derivatives	470.1944	3.1	2227.46	15756.124	↑
2-[2-[19-Acetamido-6-(3,4-dicarboxybutanoyloxy)-16,18-dihydroxy-5,9-dimethylicosan-7-yl]oxy-2-oxoethyl]butanedioic acid	Organic acid and Its derivatives	748.4024	4.9	4613.926	11399.068	↑
Tyr-Cys	Amino acid and Its metabolites	267.0797	3.6	4899.754	17874.42	↑
Isopentenyl pyrophosphate	Organic acid and Its derivatives	491.0096	2.4	30169.798	3036.56	↓
D-Ribulose 1,5-bisphosphate	Organic acid and Its derivatives	290.97	2.4	7722.868	1595.424	↓
Pranlukast	Heterocyclic compounds	480.1593	3.2	10.002	500.556	↑
Ergothioneine	Organic acid and Its derivatives	230.0924	1.9	108.26	2282.438	↑
Pantoprazole sulfide	Benzene and substituted derivatives	366.0741	3.0	2361.516	22199.344	↑
2,5-Dihydroxy benzoic acid	Benzene and substituted derivatives	153.0196	2.5	335692.638	16119.478	↓
1-Palmitoyl-2-glutaryl-sn-glycero-3-phosphocholine	Glycerophospholipids	608.3547	6.9	57499.978	28138.18	↓
FFA（18:4）	Fatty acid	277.216	7.6	23857.456	11397.334	↓
4-（Aminomethyl）benzoic acid	Organic acid and Its derivatives	210.0768	1.7	31.002	1768.68	↑
D-Arabinose	Others	185.022	0.7	30259.404	164186.404	↑

Table S3 Continuation

Metabolin	Category	m/z	RT/min	Relative conte CK	Relative conte After appressorium formation	variety
4,5-Dihydroxy phthalic acid	Benzene and substituted derivatives	197.0131	2.6	7361.334	951.02	↓
Hexanolamino PAF C-16	Glycerophospholipids	578.4147	6.9	148766.736	49547.46	↓
Lys-Phe	Amino acid and Its metabolites	400.1488	2.2	1994.034	11230	↑
Isokaempferide	Flavonoids	301.0702	5.3	6571.848	1721.77	↓
Iniparib	Benzene and substituted derivatives	326.9086	1.8	3450.05	1318.736	↓
Iridin	Flavonoids	521.1294	2.3	12632.018	45449.112	↑
Dihydrobiopterin	Heterocyclic compounds	240.1085	1.3	651.288	5138.058	↑
L-Aspartic acid	Amino acid and Its metabolites	265.0648	2.8	2279.844	34840.034	↑
Miltefosine	Organic acid and Its derivatives	430.3019	2.1	1063.626	6732.296	↑
DL-Dopa	Amino acid and Its metabolites	232.0283	1.2	39.954	702.104	↑
Umbelliferone	Coumarins	293.0572	3.9	4422.552	16688.606	↑
Lincomycin	Heterocyclic compounds	429.1977	2.1	112676.262	19413.462	↓
15(S)-Fl↑rostenol	Fatty acid	457.1796	2.1	6475.784	2011.036	↓
2-[4-[3-[3,4-Dihydroxy-4-(hydroxymethyl)oxolan-2-yl]oxy-4,5-dihydroxy-6-(hydroxymethyl)oxan-2-yl]oxyphenyl]-7-hydroxy-2,3-dihydrochromen-4-one	Organic acid and Its derivatives	550.1551	1.9	2927.104	21733.47	↑
Oxprenolol	Benzene and substituted derivatives	304.1298	2.3	649.372	1430.992	↑
(2R,3S,4S,5R,6S)-2-[[(2S,3R,4R)-3,4-Dihydroxy-4-(hydroxymethyl)oxolan-2-yl]oxymethyl]-6-(3,4,5-trimethoxyphenoxy)oxane-3,4,5-triol	Benzene and substituted derivatives	501.164	2.3	16949.13	92636.94	↑
3-Hydroxydodecanoic acid	Organic acid and Its derivatives	215.1649	6.8	5081.192	17587.142	↑
N-[2-[[[[(2S,3R)-2-(Hexadecanoylamino)-3-hydroxy-4-octadecenyl]oxy]hydroxyphosphinyl]oxy]ethyl]trimethylaminium	Sphingolipids	703.5731	7.3	1164.744	11149.788	↑
Oleragenoside	Steroids	847.4181	4.9	1707.88	823.546	↓
1,2-Dioleoyl-sn-Glycero-3-Phosphocholine	Glycerophospholipids	786.5986	7.5	23655.85	10466.176	↓
Indole-3-acetamide	Heterocyclic compounds	219.0769	2.9	4634.4	15021.226	↑
cis-11-Methyl-2-dodecenoic acid	Fatty acid	230.211	5.4	310.528	33.228	↓
1-O-trans-Cinnamoyl-beta-D-glucopyranose	Benzene and substituted derivatives	345.0754	2.6	86.842	451.45	↑
Val-Asp	Amino acid and Its metabolites	341.0986	2.4	20.32	1050.656	↑
L-tyrosine methyl ester 4-sulfate	Amino acid and Its metabolites	274.0383	2.6	20282.906	92445.994	↑

Table S3 Continuation

Metabolin	Category	m/z	RT/min	Relative conte		variety
				CK	After appressorium formation	
N-Palmitoylsphingomyelin	Sphingolipids	703.5734	6.6	4094.658	30265.328	↑
Gelsemin	Alkaloids	361.1292	2.5	5086.044	31256.074	↑
S-(5-Adenosy)-L-Homocysteine	Nucleotide and Its metabolites	384.1415	1.8	5345.204	936.516	↑
Luteolin	Flavonoids	287.0548	4.8	1370.568	4346.038	↓
Syringin	Heterocyclic compounds	407.1309	3.0	3089.284	832.144	↓
Isoquercitrin	Flavonoids	463.0914	3.0	1675.808	5693.72	↑
Melibiose	Carbohydrates and Its metabolites	387.118000000001	2.2	2361.392	12392.374	↑
Loganic acid	Organic acid and Its derivatives	375.1273	2.0	22168.372	70171.318	↑
2,6-Dihydroxy benzoic acid	Benzene and substituted derivatives	781.1651	6.3	5543.608	13033.422	↑
9,12-Octadecadiynoic Acid	Fatty acid	321.2058	4.2	3501.002	1412.464	↓
L-Aspartyl-L-phenylalanine	Amino acid and Its metabolites	279.0979	2.4	1018.47	2381.316	↑
Chrysoeriol	Flavonoids	299.0547	5.3	13303.626	2613.19	↓
Sambutoxin	Terpenoids	436.2814	6.9	63249	132916.652	↑
Cysteinyl-glutamate	Amino acid and Its metabolites	230.211	5.4	3401.68	1661.036	↓
Avocadyne 1-acetate	Fatty acid	309.2423	7.5	76454.88	34653.918	↓
Epigallocatechin gallate	Flavonoids	458.0856	2.1	2143.174	4566.618	↑
12,13-DiHOME	Fatty acid	279.2315	6.9	4006.952	713.842	↓
Iloperidone	Ketones	444.2331	1.2	13745.368	4196.212	↓
AICA ribonucleotide	Nucleotide and Its metabolites	337.0535	2.6	2383.066	798.424	↓
Adrenochrome	Heterocyclic compounds	224.0556	1.4	3896.426	8468.086	↑
Fenofibric acid	Benzene and substituted derivatives	317.0535	1.0	278253.07	137458.644	↓
Manghaslin	Flavonoids	755.2032	3.1	27356.9	6907.318	↓

Note: "m/z" represents the mass-to-charge ratio; "RT" denotes the retention time. ' ↑ ' indicates that the compound is significantly up-regulated during the formation of *Metarhizium* appressorium, and ' ↓ ' indicates that the compound is significantly down-regulated during the formation of *Metarhizium* appressorium. It should be noted that the compound identification process was based solely on pseudomolecular ions, and no MS/MS data were considered.
